# Controlling cortisol excess and comorbidities in Cushing’s syndrome with osilodrostat

**DOI:** 10.1007/s11102-025-01605-1

**Published:** 2025-12-21

**Authors:** Maria Fleseriu, John Newell-Price, Mônica R. Gadelha, Beverly M.K. Biller

**Affiliations:** 1 Pituitary Center, Departments of Medicine and Neurological Surgery, Oregon Health & Science University, Portland, OR USA; 2 School of Medicine and Population Health, University of Sheffield, Sheffield, UK; 3 Neuroendocrinology Research Center, Endocrinology Section, Medical School and Hospital Universitário Clementino Fraga Filho, Universidade Federal do Rio de Janeiro, Rio de Janeiro, Brazil; 4 Neuroendocrine and Pituitary Tumor Clinical Center, Massachusetts General Hospital, Boston, MA USA

Osilodrostat, a potent 11β-hydroxylase inhibitor with extensive clinical-trial and real-world data, demonstrated rapid and sustained reductions in mean urinary free cortisol (mUFC), alongside improvements in signs and symptoms of hypercortisolism and quality of life (QoL), in patients with Cushing’s syndrome (CS) [1–7].

Our recent article in *Pituitary* reported findings from the largest prospective analysis of long-term changes in blood pressure (BP) and glycemic and clinical parameters in patients with Cushing’s disease (CD) receiving long-term osilodrostat treatment [8]. To our knowledge, this is the first analysis of prospective data to assess correlations between long-term changes in BP and glycemic control and changes in the clinical characteristics of CD. Of the 210 patients included in the LINC 3 and LINC 4 studies, 82.9% had hypertension and 40.0% had diabetes at baseline. Osilodrostat led to rapid improvements in systolic and diastolic BP, and fasting plasma glucose (FPG) and glycated hemoglobin (HbA_1c_) levels, as early as week 12, which were maintained during long-term treatment (up to 72 weeks). Improvements in BP and glycemic parameters were greatest in those with high baseline systolic and diastolic BP, as well as high baseline FPG and HbA_1c_ levels. Nearly 50% of hypertensive patients and >60% of patients with diabetes experienced normalization of BP or glycemic parameters, with some able to reduce or stop their antihypertensive or antihyperglycemic medications (Fig. 1). New cases of hypertension and diabetes were rare; most patients without these comorbidities at baseline did not experience increases in BP or glycemic parameters. Notably, improvements in BP and glycemic parameters correlated with improvements in mUFC. This further strengthens the link between cortisol excess and increased cardiometabolic risk, reinforcing the importance of achieving early and complete biochemical control. These clinically meaningful cardiometabolic improvements with osilodrostat provide evidence of its impact on comorbidity burden. Although this analysis is strengthened by its large, pooled population from two pivotal Phase III studies and its 72-week long follow-up period, it is limited by its *post hoc*, descriptive design. There is a need for longer-term, independent, real-world studies to support these findings and determine whether early therapeutic intervention with osilodrostat may prevent cardiometabolic complications of hypercortisolism.

**Fig. 1:**
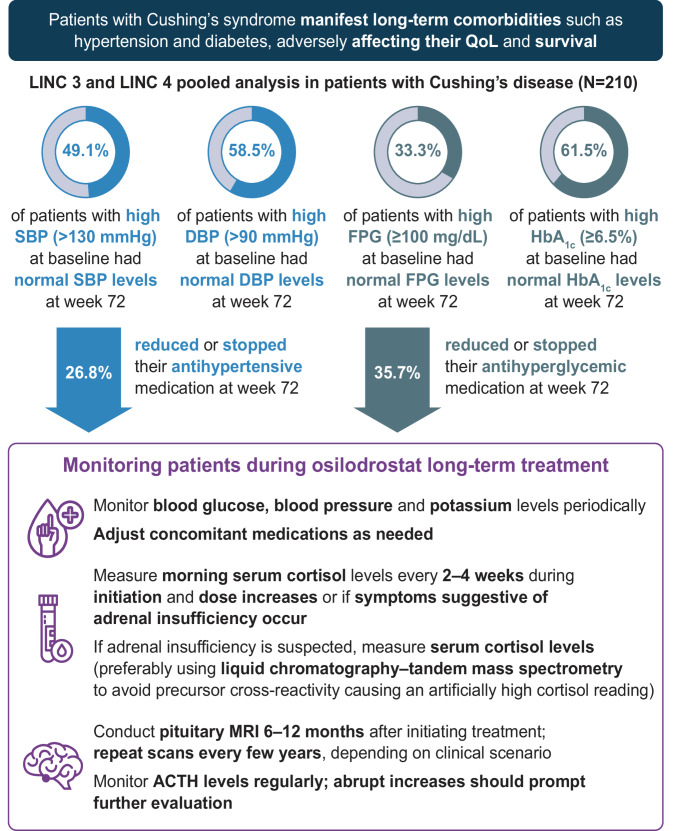
Summary of the findings from the LINC 3 and LINC 4 pooled analysis evaluating the effect of osilodrostat on blood pressure and glycemic control in patients with Cushing’s disease [8] and key considerations for monitoring patients during osilodrostat long-term treatment. ACTH, adrenocorticotropic hormone; DBP, diastolic blood pressure; FPG, fasting plasma glucose; HbA_1c_, glycated hemoglobin; MRI, magnetic resonance imaging; QoL, quality of life; SBP, systolic blood pressure

The findings from the pooled LINC 3 and LINC 4 analysis are clinically relevant as most patients with CS have hypertension at diagnosis (80–85%) and diabetes is a frequent early complication of CS [9, 10]. Another common comorbidity of CS is bone disease, with uncoupled bone formation and bone resorption leading to an increased risk of skeletal complications [11, 12]. Patients with a higher overall burden of cardiovascular morbidity, including those with hypertension and impaired glucose metabolism, are more likely to experience these fragility fractures [13]. CS is also associated with an increased risk of all cancer types compared with the general population [14]. These long-term comorbidities adversely affect QoL and survival [12, 15], with the risk of morbidity and mortality persisting, particularly in patients not in biochemical remission [15, 16]. The results from the LINC 3 and LINC 4 pooled analysis indicate that osilodrostat may reduce the treatment burden associated with long-term comorbidities in patients with CD [8].

Although surgery is recommended as first-line therapy [17, 18], the contribution of medical therapy in the management of patients with CS is increasing. Medical therapy is an option for patients with persistent or recurrent disease after surgery to remove the tumor, for those who refuse or are not eligible for surgery, for those requiring emergency treatment for severe hypercortisolism, and as a bridging therapy for those waiting for surgery or for the effects of radiation for pituitary CS [12, 17, 18]. In a recent study, adrenal steroidogenesis inhibitors were the most used medications at pituitary tumor centers of excellence [19]. With increased use of osilodrostat in clinical practice, additional practical, evidence-based guidance for tailoring treatment is needed.

Guidelines advocate a personalized treatment approach for CS, aiming for cortisol control while considering patient preferences and how comorbidities may impact patient health and QoL [17, 18]. Despite theoretical concerns regarding accumulation of mineralocorticoid precursors, new or worsening hypertension and hypokalemia were uncommon in clinical studies of osilodrostat [2, 4, 8]. However, comorbidities should be closely monitored during osilodrostat treatment as adjustments in concomitant medications are required for some patients when cortisol levels decline, including those who experience improvements in hypertension or diabetes [8].

Although the LINC studies did not specifically focus on patients with higher baseline mUFC levels [20], a higher starting dose and/or faster dose escalation may be considered in severe cases requiring urgent mUFC control. Conversely, in our clinical experience, lower starting doses and/or slower dose escalation may be considered in patients with mild mUFC elevations (>1–1.3 times the upper limit of normal). Additionally, patients of Asian origin may require lower doses than non-Asian patients because of the higher bioavailability of osilodrostat in this population [21].

Adverse events (AEs) related to adrenal insufficiency (AI) are expected with most treatments for CS, especially steroidogenesis inhibitors. In the LINC trials, hypocortisolism-related AEs (as determined by investigators) were mostly mild to moderate and manageable predominantly with temporary interruption and glucocorticoid therapy [20]. In clinical practice, differentiating between glucocorticoid-withdrawal syndrome (GWS) and AI can be challenging because of overlapping symptoms, including musculoskeletal discomfort, fatigue, weight loss and anorexia [22]. GWS is associated with abrupt reduction in cortisol levels after prolonged pathological elevation and may be a low-grade inflammatory state caused by upregulation of cytokines and prostaglandins [22, 23]. Physical deconditioning, mood/cognitive disturbance and hypersomnia suggest a GWS diagnosis [23]. AI is associated with true low cortisol levels and usually displays more severe symptoms than GWS, such as vomiting, hypotension and hypoglycemia, requiring glucocorticoid-replacement therapy [22, 23]. Biochemical assessments of cortisol levels can provide valuable insight; however, results must be interpreted in the context of the clinical presentation [23]. Where results are available in a timely manner, we recommend assessing serum cortisol levels with liquid chromatography–tandem mass spectrometry in patients with suspected AI. Alternative cortisol assays may be misleading because of potential cross-reactivity in some immunoassays of precursors (11-deoxycortisol) with cortisol [17], giving a false impression of higher circulating cortisol values with potential for inappropriate dose escalation. Clinical decisions may be required before laboratory results are available; as AI can be life threatening, we recommend treating patients with suspected AI or GWS with glucocorticoid-replacement therapy (particularly if the patient has a sudden drop in BP or increased heart rate upon standing). Treatment of confirmed AI includes replacement or stress-dose glucocorticoids and fluid repletion, according to treatment guidelines [24, 25]. We recommend monitoring patients receiving osilodrostat for hypocortisolism-related AEs by assessing morning serum cortisol levels every 2–4 weeks, especially at treatment initiation, up-titration and during instances of increased cortisol demand (physical/psychological stress or changes in concomitant medications that may affect osilodrostat exposure).

As AI may occur after the patient has received osilodrostat for some time [20], regular biochemical and clinical assessments for the duration of treatment and during temporary dose interruptions are important, alongside comprehensive patient education to raise awareness, so that AEs can be detected early and treatment initiated to prevent severe acute adrenal crises. Risk of prolonged AI has also been noted in several reported cases [26]. Patients with adrenocorticotropic hormone (ACTH)-dependent CS treated with osilodrostat may experience adrenal shrinkage, with or without AI, and require lower doses to remain biochemically controlled [27].

How to monitor potential adenoma growth in patients with CD treated with adrenal steroidogenesis inhibitors remains controversial. Although mean ACTH levels increased steadily from baseline in the LINC trials, no correlations were identified with total daily osilodrostat dose or tumor volume over time [20]. Nevertheless, in our opinion, ACTH levels should be monitored regularly for progressive, sustained increases in ACTH, excluding the expected rise and plateau at the start, when osilodrostat is initiated. Pituitary magnetic resonance imaging 6–12 months after initiating treatment and repeat scans every few years thereafter, depending on the clinical scenario, has been recommended [17]. If progressive adenoma growth is identified, medical treatment that is not pituitary directed should be withheld and the management plan reassessed. Longer-term data are needed to determine any attributable risk of tumor growth with osilodrostat use.

Better markers of control are needed to optimize the management of patients with CS. In clinical practice, mUFC is frequently used but has a high degree of variability, with multiple sample collections recommended [17]. Late-night salivary cortisol (LNSC) is more likely to show abnormal findings before mUFC when CS is developing or recurring, potentially allowing for earlier intervention [17]. However, LNSC is more sensitive to disruption of circadian rhythm and takes longer to normalize than mUFC with treatment, so more regular assessment of multiple LNSC samples over time may provide more accurate results [28]. A recent pooled analysis of LINC 3 and LINC 4 demonstrated a moderate correlation between LNSC and mUFC during osilodrostat treatment [28]. Patients with control of both LNSC and mUFC generally exhibited greater improvements in cardiovascular and metabolic-related parameters than those with only mUFC control or uncontrolled LNSC and mUFC [28]. Treatments should therefore aim to normalize both mUFC and LNSC for optimal patient outcomes, provided that morning cortisol levels are maintained in the normal range. A recent study used osilodrostat once daily in the evening as a ‘reverse circadian’ dosing schedule, which preserved morning cortisol levels while lowering abnormal late-night/overnight levels [29]. More data are needed on this interesting approach. Other potential novel markers of control that do not rely on cortisol measurements are being identified, including changes in biomarkers (growth-differentiating factor 15 and osteocalcin), metabolomic profiles, micro-RNA, gene expression and epigenetics [30]. Future assessment of biochemical control may incorporate a comprehensive evaluation of these novel biomarkers, cortisol levels and changes in clinical signs and symptoms of disease.

There is a wealth of data for osilodrostat from clinical trials, including the pooled analysis of >200 patients from the LINC 3 and LINC 4 Phase III studies [8], real-life studies and our clinical practice experience. Together, these demonstrate that osilodrostat achieves mUFC normalization, leads to clinical improvements in comorbidities and improves QoL in most patients with CS. Balancing efficacy with safety is important, and for most patients, slow up-titration is needed. However, patients with severe CS may benefit from a ‘block-and-replace’ regimen with higher osilodrostat starting doses. Personalizing the management of patients with CS is key to optimizing outcomes and improving patient satisfaction. Future research should explore the integration of osilodrostat into personalized treatment algorithms and its real-world impact on long-term cardiovascular and metabolic outcomes.

## Data Availability

No datasets were generated or analyzed during the current study.
